# Neuropathogenesis of Old World Alphaviruses: Considerations for the Development of Medical Countermeasures

**DOI:** 10.3390/v17020261

**Published:** 2025-02-14

**Authors:** Alyssa M. Lantz, Victoria K. Baxter

**Affiliations:** Texas Biomedical Research Institute, San Antonio, TX 78227, USA; alantz@txbiomed.org

**Keywords:** Old World alphaviruses, chikungunya virus, neuropathogenesis, neurological disease, vaccines, treatments

## Abstract

Chikungunya virus (CHIKV) and other alphaviruses that primarily induce arthritogenic disease in humans, known as “Old World” alphaviruses, present an emerging public health concern as geographic ranges of mosquito vectors expand due to climate change. While a vaccine against CHIKV has recently been approved by several countries in North America and Europe, access to effective preventative countermeasures against disease induced by Old World alphaviruses remains elusive for the most vulnerable populations. Furthermore, treatment options continue to be limited to supportive care. Atypical neurological disease manifestations caused by Old World alphaviruses, which make up as many as 25% of the cases in some CHIKV outbreaks, present special challenges when considering strategies for developing effective countermeasures. This review focuses on Old World alphaviruses, specifically CHIKV, Ross River virus, O’nyoug-nyoug virus, and Mayaro virus, concentrating on the atypical neurological disease manifestations they may cause. Our current understanding of Old World alphavirus neuropathogenesis, gained from human cases and preclinical animal models, is discussed, including viral and host factors’ roles in disease development. The current state of alphavirus preventatives and treatments, both virus-targeting and host-directed therapies, is then summarized and discussed in the context of addressing neurological disease induced by Old World alphaviruses.

## 1. Introduction

Alphaviruses are positive-sense, single-stranded RNA viruses in the *Togaviridae* family. Most clinically important alphaviruses are transmitted by mosquitoes. Due to mosquito vectors expanding to new geographical regions, alphaviruses represent a re-emerging public health concern [1,2,3]. In particular, the alphavirus chikungunya virus (CHIKV) has been declared a high-risk threat to public health, as the geographic extent of its primary vectors, *Aedes aegypti* and *Aedes albopictus*, has increased as global temperatures have risen [4]. Reports of CHIKV cases and deaths have increased four-fold from 2022 to 2023 [2]; however, these statistics are likely underestimated due to inconsistent, inadequate surveillance and misdiagnosis [4,5].

Alphaviruses are traditionally categorized as “New World” or “Old World” based on historical geographical origins and correlate with natural disease typically induced in humans [6]. New World alphaviruses, which include eastern equine encephalitis virus, western equine encephalitis virus, and Venezuelan equine encephalitis virus, typically induce neurological disease and pathology. Old World alphaviruses, in addition to CHIKV, include, but are not limited to, Ross River virus (RRV) [7], O’nyoug-nyoug virus (ONNV) [3], and Mayaro virus (MAYV) [8,9]. These alphaviruses tend to cause a more systemic disease characterized by headache, fever, and malaise, along with arthritis and arthralgia [10]. However, Old World alphavirus infection may also result in neurological disease and pathology depending on the virus.

Understanding the pathogenesis of alphaviruses is critical to developing effective countermeasures. Here, we review the host and viral factors’ roles in Old World alphavirus pathogenesis, specifically focusing on atypical neurological disease manifestations. We also discuss how the nature of neurological disease induced by Old World alphaviruses presents additional challenges when designing treatment strategies.

## 2. Neurological Disease Induced by Old World Alphaviruses

### 2.1. Epidemiology and Vectors

CHIKV was one of the first isolated Old World alphaviruses identified to cause clinical disease in humans [11]. CHIKV was first identified in 1952 in Tanzania [12]. For half a century following the first large outbreak in 1952, CHIKV was limited to occasional outbreaks in Africa and Asia [13]. However, CHIKV evolved and adapted to a new vector, resulting in a large-scale outbreak in islands across the Indian Ocean, including La Réunion, Seychelles, Mauritius, Madagascar, and Mayotte in 2005–2006 [14]. Three phylogenetic lineages of CHIKV have now been identified: East, Central, and South African (ECSA), West African, and Asian. As of 2024, CHIKV has spread globally, with over 3.7 million cases reported in the Americas over the last decade, including the United States [13]; an additional few cases have been documented in Europe.

ONNV and MAYV are Old World alphaviruses that, thus far, are relatively limited geographically. ONNV was first isolated during an epidemic in 1959 in Uganda [15], during which over 2 million cases were reported over three years. Since then, the virus has spread across Central, East, and West Africa but has not been identified outside of the African continent, except for some imported cases in Canada and Germany [3]. However, the number of cases is likely under-represented. A close evolutionary relationship exists between CHIKV and ONNV [3], with 89% genome conservation [16]. MAYV was first identified in five patients with febrile illness in Trinidad and Tobago in 1954 [17]. Since then, outbreaks have been restricted to Central and South America, usually in tropical rainforests [9]. Imported cases have been identified in non-forested areas, including North America and Europe [18]. As it is transmitted by the same vector as CHIKV, possible urbanization of MAYV presents an emerging public health concern [9,18,19].

RRV is one of the most common arboviruses in Australia [20,21]. It was first isolated in mosquitoes in Queensland, Australia, in the 1950s and was first isolated from humans in the early 1970s [20,22]. The primary reservoir host is marsupials, specifically kangaroos and wallabies [20,23,24,25]; however, some evidence suggests human-to-human transmission can occur without an intermediate mosquito host, though this finding is not conclusive [26]. RRV is endemic in Australia and Papua New Guinea but has been detected elsewhere, including French Polynesia and American Samoa [7].

Other Old World alphaviruses, including Sindbis virus (SINV) and Semliki forest virus (SFV), generally are considered non-pathogenic but have been associated with sporadic cases of fever, myalgia, and arthralgia [12]. SINV is widespread through Europe, Asia, and Africa, but most cases in humans are reported in Scandia and caused by Ockelbo, Pogosta, or Karelin SINV strains [12,26]. SFV is also usually non-pathogenic in humans, causing mild fever and arthralgia, but a case of laboratory-acquired SFV infection leading to fatal encephalitis has been reported [27,28]. These alphaviruses are commonly used as models for alphavirus neuropathogenesis in mice and will be discussed later in the review [12].

Old World alphaviruses typically maintain a sylvatic cycle, transferring between mosquito vectors and vertebrate hosts [29]. However, transmission from indiscriminate mosquitoes to humans occasionally occurs, and in more extreme situations, such as seen with CHIKV, a second urban cycle between mosquitoes and humans is established. The primary vector of CHIKV in urban areas is *Ae. aegypti* [30]. However, in the early 2000s, a mutation (E1 A226V) identified during an ECSA-lineage outbreak in the Indian Ocean led to the virus adapting to *A. albopictus* [14,31]. ONNV is unique compared to other alphaviruses as it is transmitted by *Anopheles* mosquito species, which are vectors of *Plasmodium* spp. that cause malaria [3]. Arboreal *Haemagogus janthinomys* mosquitoes primarily transmit MAYV, but urbanization of this virus is possible, as *Ae. aegypti* can also transmit MAYV [9,18,19]. One case in Haiti involving an 8-year-old boy showed co-infection of dengue virus (DENV) and MAYV [32], which suggests that *Ae. aegypti* may have been the mosquito vector. RRV is considered a vector generalist, as it can be transmitted by over 40 species of mosquitoes [7,19,33], including *Ae. aegypti* and *Ae. albopictus*. With the expansion of mosquitoes and the rise in global temperature, it is plausible for RRV to spread worldwide.

### 2.2. Neurological Disease Manifestations and Sequelae

CHIKV, ONNV, MAYV, and RRV are a part of the Semliki Forest virus complex and induce similar disease manifestations. Typical acute Old World alphavirus infections are self-limiting and characterized by fever, polyarthralgia, myalgia, and rash, with significant symptom overlap [10]. Small joints of the extremities, including hands, wrists, fingers, and knees, are most likely to be affected. Following acute infection, patients can develop chronic arthritic symptoms that can last months to years following initial infection [3,18,34,35]. Some persistent symptoms include fatigue, joint tenderness, joint and muscle pain, and musculoskeletal stiffness [12]. However, atypical neurological sequelae are described following infection with some of these viruses, especially CHIKV. Table 1 summarizes case reports of neurological disease currently for Old World alphaviruses.

Between 7 and 25% of CHIKV cases during outbreaks have been reported to include neurological disease symptoms [12,38,48,49]. Of the three phylogenetic CHIKV clades, ECSA strains have been more closely associated with neurological symptoms [12,50]. However, neurovirulence has also been reported in individuals infected with Asian strains [50], and genotyping is rarely done in clinical settings to distinguish strains from different phylogenetic lineages. Encephalitis and peripheral neuropathologic conditions such as Guillain–Barre syndrome (GBS) are the most commonly reported neurological disease manifestations [38,49]. Pathological changes in the central nervous system (CNS) tissue include perivascular and parenchymal infiltration of mononuclear cells, microgliosis, and demyelinating lesions [41,51,52]. Survivors of the initial course of neurological disease are often left with permanent neurological sequelae that result in significant lifelong disability [38,48,53].

ONNV typically induces a clinical disease similar to CHIKV infection, though without acute edema in joints [3,54]. No known neurological complications have been associated with ONNV infection [3], but in vitro studies have shown Schwann cells are permissive to infection, and A129 and *Stat1^−/−^* mice are susceptible to occasional neuroinvasion [55]. MAYV manifests as a non-specific, self-limited acute febrile illness that is often misdiagnosed for DENV or CHIKV; however, fever can last up to ten days, distinguishing the virus from other arboviruses that tend to circulate in the same regions [9,18]. MAYV can infect human neural progenitor cells, pericytes, and astrocytes in vitro [56], and one case of neurological disease has been reported in humans [45]. A 47-year-old male patient in French Guiana diagnosed with acute meningoencephalitis after a five-day history of frontal headaches, photophobia, meningeal stiffness, fatigue, aphasia, and confusion was positive for MAYV IgM [45]. Since ONNV and MAYV are genetically similar to CHIKV, both viruses theoretically have the propensity to evolve and induce neurological disease [57]. However, viral evolution is influenced by many factors, including, but not limited to, cycling between different host species, climate, and vector geographical range [58]; therefore, it is currently unclear whether these alphaviruses will evolve to induce neurological disease based solely on genetic similarity to CHIKV.

While most cases of RRV infection are asymptomatic, when present, symptoms include rash, fever, flu-like illness, lethargy, myalgia, arthralgia, and joint stiffness [20,33,59]. Symptoms, mainly arthralgia and joint stiffness, may persist for months past infection, with only 27% of patients in one study recovering fully by six months [59]. Atypical sequelae have been reported, including neurological manifestations such as encephalitis and meningitis [33]. However, definitive proof that RRV is responsible for the few reported cases of neurological disease has not been made [33,47]. One paper documented a potential imported case where a 33-year-old male had traveled to Australia during an RRV outbreak and was bitten by mosquitoes [47]. He presented with encephalitis at the hospital in his home country, tested positive for RRV IgM and IgG, and had persistent hip and shoulder pain weeks after discharge, supporting the possibility that RRV also caused his neurological symptoms.

### 2.3. Using Animal Models to Study Alphavirus Neuropathogenesis

The limited accessibility and minimally regenerative nature of the CNS prevent alphavirus neuropathogenesis studies from being performed in humans and require animal models, especially when evaluating disease pathogenesis. CHIKV encephalitis has been studied in multiple animal models, including zebrafish [60,61] and macaques [62,63]. However, mice are a valuable tool because of their comparable immune system to humans, genetic manipulability, and lower cost than macaques. Several papers investigating CHIKV neuropathogenesis in mouse models have been published [50,64,65,66,67,68]. Most studies have used neonatal or immunocompromised mice, limiting the translatability of the findings to susceptible human populations. Young adult (4–6 week old) C57BL/6J (B6) mice have been identified as a potential immunocompetent and neurodevelopmentally appropriate mouse model to study direct CNS infection with CHIKV [69,70]; mice in this age range are neurodevelopmentally equivalent to a one- to two-year-old child, an especially vulnerable age for developing neurological disease following alphavirus infection [38,40]. Attempts to achieve CNS infection following peripheral inoculation have generally been unsuccessful in adult B6 mice; however, one group reported successful brain infection following subcutaneous footpad injection with a CHIKV strain isolated from Delhi in 2010 [71,72]. While arthritogenic disease pathogenesis and host immune responses have been studied in mouse models for MAYV [73,74], ONNV [55,75], and RRV [76,77], no publications describing animal models of neurological disease induced by any of these viruses have been released to date. However, a paper describing subcutaneous infection of three rhesus macaques with MAYV showed the virus could disseminate to the CNS, though neurological signs were not identified in any of the animals [78].

As mentioned previously, while SINV and SFV do not induce significant human disease, particularly neurological complications, from natural infection, these viruses have played an outsized role in understanding neurological disease caused by alphaviruses using mouse models. The naturally occurring SINV strain AR339 can disseminate to the CNS effectively, and a correlation has been found between age and severity of SINV AR339-induced disease, with neonatal mice more susceptible to lethal disease than weanling mice [79,80]. SINV has been adapted to be neurotropic in mice, providing a valuable, well-characterized model to study alphavirus encephalomyelitis [81,82]. A neuro-adapted SINV strain, NSV, was created by serially passaging the AR339 strain six times in neonatal and weanling mice via intracerebral inoculation [83]. NSV induces 100% mortality in susceptible mice of all ages [12,83]. SINV has been shown to enter the CNS through axonal transport and disseminate throughout the brain and spinal cord, where it infects neurons and can lead to neuronal damage and death [12].

While SFV typically causes mild fever, myalgia, and arthralgia in humans, in mouse models, infection induces meningoencephalomyelitis with demyelination [84,85]. The avirulent strain A7(74) produces a transient viral infection in mice. While BALB/c mice do not develop clinical disease, severe combined immunodeficiency (SCID) mice develop paralysis and moderate mortality by 5 days post-infection (dpi) [12,86]. The virus enters the CNS by infecting vascular endothelial cells [87], and while SFV can infect neurons, in contrast to SINV, oligodendrocytes represent the primary target cell in the CNS [12].

Several approaches, including microarray, RNA sequencing, and candidate gene studies, have been developed in animal models to study different factors that affect alphavirus pathogenesis, particularly host genetics’ role in disease [88]. In addition, newer systematic approaches are now being used to understand how heterogeneous populations react to alphavirus infections. One study that screened eight different inbred mouse strains and compared disease severity following SFV intraperitoneal infection found that while all strains developed encephalitis, only three showed definitive signs of demyelination [89]. Another study comparing BALB/c and B6 mice infected with SINV NSV identified a locus on chromosome 2, Nsv1, associated with viral load, development of paralysis, and death [88,90].

While mouse models have played a critical role in understanding alphavirus neuropathogenesis, their use presents multiple limitations. As discussed above, limited data exist on successful alphavirus neuroinvasion following peripheral infection in adult B6 mice. In most studies, investigators infect mice by intracranial inoculation, which is an unnatural route of infection and limits translation to the natural course of disease in humans. Because alphaviruses are transmitted by mosquitoes through peripheral infection, subcutaneous inoculation represents a more natural route of infection by which to understand mechanisms of neuroinvasion and develop potential medical countermeasures. Furthermore, as described above, the common use of neonatal or immunocompromised mice in alphavirus neuropathogenesis studies presents an additional limitation to translatability. An immunocompetent mouse is needed to fully understand immune-induced pathology and develop effective host-directed interventions. In addition, neonatal mice are neurodevelopmentally equivalent to a third trimester fetus [69], which limits their translatability to both adult humans and children less than two years of age, one of the most vulnerable populations to neurological alphavirus infection.

## 3. The Role of Host-Pathogen Interactions on Alphavirus Pathogenesis

**Figure 1:**
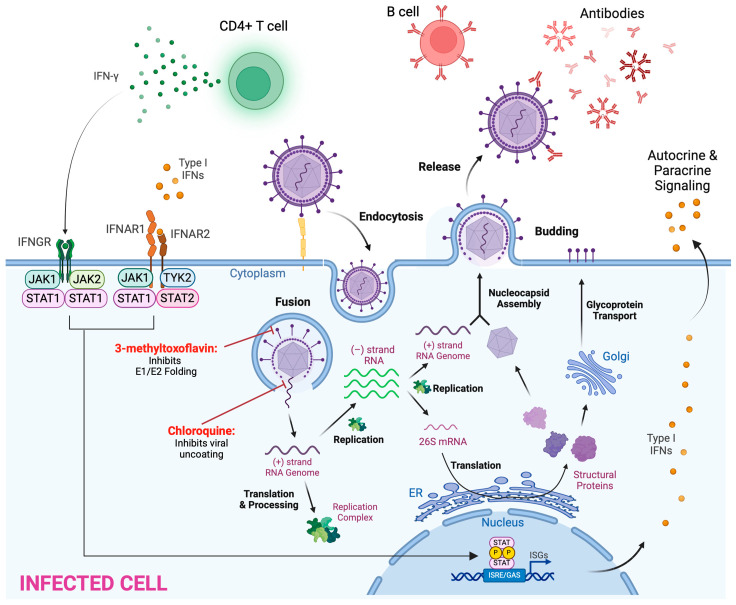
Alphavirus replication in the host cell, including steps targeted by antivirals, and the antiviral response mounted by the host. This figure was created using biorender.com.

Figure 1 summarizes select aspects of the anti-alphaviral response discussed in this section.

### 3.1. Viral Factors

Alphaviruses are made up of structural (sP) and non-structural (nsP) proteins critical for promoting virus replication while hindering antiviral cellular responses. sPs make up the virions and include the capsid protein and glycoproteins. The E1 and E2 glycoproteins facilitate attachment and entry into host cells and are the primary targets for neutralizing antibodies [35]. As mentioned previously, a single point mutation in the E1 glycoprotein acquired by CHIKV (A226V) has allowed the virus to utilize *Ae. albopictus* in addition to *Ae. aegypti* as transmission vectors [14]. The capsid protein is critical for forming viral particles that encapsulate the viral genome [35]. nsPs contain the core enzymes that are necessary for viral replication but also combat host antiviral mechanisms [35]. nsP1 and nsP2 disrupt immune signaling pathways, which inhibit antiviral gene expression [35]. nsP3 is critical for viral replication and, as the least conserved nsP of the alphavirus proteome, has a high potential for acquiring mutations beneficial to the virus [3,35]. An extensive review of the viral factors mediating CHIKV virulence has been published by Freppel et al. [35].

Data on how viral factors affect neuroinvasion during CHIKV, ONNV, MAYV, and RRV infection are limited. However, during SINV infection in mice, while multiple mechanisms are important for neuroinvasion, the E2 glycoprotein is the primary driver [91,92]. Furthermore, during SFV infection, E2 is important for crossing the blood-brain barrier (BBB) through interactions with glycosaminoglycans (GAGs) [93]. Therefore, it is reasonable to assume that mutations in the sPs of other alphaviruses could enhance susceptibility to neuroinvasion, thus potentially leading to neurological disease.

Alphaviruses can counteract different antiviral cellular factors and immune mechanisms, thus increasing virulence [94,95]. Type I interferons (IFNs) are crucial for controlling initial viral infection, making the IFN pathway a common target for viruses [95]. The cyclic GMP-AMP synthase (cGAS) stimulator of IFN genes (STING) pathway plays an integral part in the innate immune system by detecting double-stranded DNA (dsDNA), which leads to stimulation of IFN-stimulated genes (ISGs) and expression of type I IFNs [95]. During the first few hours of CHIKV infection, cGAS expression is downregulated by viral capsid protein, and viral nsP1 interacts with STING to decrease type I IFN expression, resulting in higher viral loads and delayed clearance [94,95]. cGAS also plays a role in controlling ONNV, MAYV, and RRV infection [3,96], suggesting other alphaviruses may have developed similar innate immune evasion strategies.

With CHIKV, we can compare virus strains belonging to different phylogenetic lineages to understand further how viral genetic diversity affects neuroinvasion. Compared to an Asian CHIKV strain isolated in the 1960s, an ECSA-lineage CHIKV strain isolated from the 2006 La Reunion outbreak was found to induce increased foot swelling associated with infiltration of mononuclear cells and higher serum proinflammatory cytokine concentrations in mice [97]; no virus was detected in mouse brains during the study. However, another study demonstrated that CHIKV-induced neurological disease in young adult B6 mice was strain dependent, with an ECSA-lineage CHIKV isolated during an outbreak in Sri Lanka showing increased disease severity compared to an Asian CHIKV isolated during an outbreak in the Caribbean island of San Martin [70]. In contrast, when comparing two CHIKVs isolated in Malaysia, the Asian-lineage strain showed higher mortality and increased expression of apoptotic genes in suckling mice compared to the ECSA-lineage strain [50]. Further work is needed to dissect what viral and host factors drive CHIKV strain-dependent disease severity, which will allow for better identification of vaccine targets and treatment strategies.

### 3.2. Host Factors

In addition to viral factors, multiple host factors, including age [79], immune pathways [83,98,99,100], and host genetics [90], have been found to affect alphavirus neuropathogenesis. In humans, the nature of neurological disease developed by patients infected with CHIKV tends to depend on the individual’s age, with young children and elderly adults with comorbidities more likely to develop CNS disease and middle-aged adults tending to develop autoimmune-mediated peripheral neuropathies [38]. During a CHIKV outbreak in Honduras in 2015, of the children less than one year of age admitted to a hospital with a CHIKV diagnosis confirmed by PCR, 24% developed neurological disease, such as meningoencephalitis and seizures [40]; in contrast, only 7% of adults admitted to the same hospital with a CHIKV diagnosis developed neurological complications [41]. Similar enhanced age-related susceptibility to CHIKV-induced mortality has been observed in mice [70]. ONNV and MAYV also show a correlation between age and mortality in mice. Only 50% of 6-week-old *Stat1^−/−^* mice survive ONNV infection, compared to 100% of 12–16-week-old mice [55]. For MAYV, while 100% mortality has been observed in 6-day-old SV129 mice, 8-week-old mice show 100% survival [74]. In addition, RRV-induced myositis shows similar age dependence in mice [101]; however, this does not reflect clinical findings with RRV infection, where individuals under the age of 18 are primarily asymptomatic [7,33].

The human population is heterogeneous, and host genetics are known to affect susceptibility to viral infections, including alphaviruses [102]. Genetic mutations in cell surface receptors can alter viral entry into target cells [88], and markers associated with altered susceptibility to infection and disease outcome with CHIKV and other Old World alphaviruses include HLA, TLR-3, TLR-7, TLR-8, MXRA8, and CD209 (DC-SIGN) [3,9,35,88,102]. While studies evaluating the role of genetics on alphavirus-induced neurological disease are limited, encephalomyelitis following RRV infection has been reported in neonatal BALB/c mice, but not adult B6 mice; the extent to which this altered susceptibility is driven by age versus mouse strain is unclear [76,103]. In contrast, disease susceptibility differences between these two mouse strains have been more definitively seen following SINV NSV and SFV infection [84,100,104,105]. BALB/c and B6 mice infected with the SFV mutant M9 showed differences in disease manifestations, with BALB/c mice showing more severe myelin vacuolation and demyelination, but B6 mice demonstrating higher mortality [84]. Natural differences in the immune response between strains could explain this variation in clinical outcome; BALB/c mice tend to skew towards a Th2 immune response [106], while B6 mice tend to skew towards a Th1 response [107]. These studies highlight host genetics’ roles in disease outcomes during infection with alphaviruses. It is important to note that both neuroinvasion and neurovirulence are complex processes and involve the interplay of both host and viral factors. Future work should integrate host systems genetic approaches, such as those described above, with methods evaluating viral genetics to understand these complex host-pathogen interactions fully.

Co-infections in the host can also play a significant role in alphavirus-induced disease outcomes. Mechanisms include excessive immune activation and widespread inflammation that can spread to the CNS, damaging neurons and impeding their functionality [12,94]. In addition, co-infections can affect the permeability of the BBB, and interactions between different pathogens may increase alphavirus virulence [94]. The public health impact of CHIKV is further complicated in that CHIKV and flaviviruses DENV and ZIKV tend to circulate in the same geographic regions, are all transmitted primarily by *Ae. aegypti*, and share similar symptoms, which can lead to misdiagnosis [2,5,108,109]. Therefore, it would benefit the field to investigate co-infections with other viruses endemic in the same area as these Old World alphaviruses to further understand their interactions and how they can affect neurological disease.

### 3.3. Immune Responses and Immunopathogenesis

Most of our understanding of the immune system’s role in alphavirus neuropathogenesis comes from studies using SINV and SFV in mice. Both the innate and adaptive immune responses are known to be critical for alphavirus clearance in the CNS. The innate immune system detects CHIKV through pattern recognition receptors (PRRs), which drive the priming of the adaptive immune response and promote inflammatory responses [35,94]. Type I IFNs play a significant role in protecting against alphavirus infection, with mice deficient in type I IFN pathways showing increased viral titers and disease severity [67,110,111]. Studies have shown virus in the brains of ONNV-infected *Stat1^−/−^* and A129 mice [55], suggesting that type I IFN signaling plays a role in protecting against ONNV neuroinvasion [67,73,74]. The innate immune response is also crucial for restricting MAYV infection, as adult type I IFN receptor-deficient (*Ifnar^−/−^)* mice develop severe disease [74]. Macrophages play a role in RRV pathology, and muscle damage is inhibited when macrophage-toxic agents are administered to RRV-infected mice [112], consistent with in vitro studies in RRV-infected striated muscle cells [113]. Furthermore, macrophages might provide a route for RRV to gain entry into the brain during infection, serving as a “Trojan horse” for neuroinvasion [114].

Clearance of alphaviruses from the CNS has been most thoroughly elucidated in mouse models of SINV infection. Clearance of infectious virus is dependent on cooperation between 1) IFN gamma (IFNγ) and 2) anti-SINV antibody [99,115]. IFNγ increases chemokines that attract B cells to the brain and promotes the skewing of IgG subclasses, aiding in viral clearance [115]. However, IFNγ also promotes neuropathology; mice deficient in the IFNγ receptor have delayed viral clearance but also show minimal pathological changes in the CNS compared to wild-type mice with intact IFNγ signaling [99]. These findings illustrate the “double-edged” sword phenomenon seen with the immune response to many viruses, including alphaviruses.

Mouse models using SINV have shown that the immune system plays a pathogenic role during infection, with T cells, especially CD4+ T cells, serving as the primary drivers of pathology in the CNS. Infiltration of immune cells into the CNS correlates with neurological disease and mortality [12,98,99]. SCID mice, which lack mature T and B cells, show minimal pathology and 100% survival following SINV NSV infection [81]. T and B cells have been shown to play a role in demyelination during SFV infection; SCID mice show robust viral replication but reduced or delayed pathology following SFV infection [86]. CD8+ T cells, but not CD4+ T cells, have been found to drive CNS damage, as depletion of CD8+ T cells reduces inflammation and eliminates demyelination [116]. CHIKV-induced joint disease is driven by CD4+ T cells [3,12,72], and neurological infection with a more neurovirulent CHIKV strain has been associated with more CD4+ T cells infiltrating the CNS compared to less neurovirulent CHIKV strains [70], suggesting a similar pathogenic mechanism occurs during CHIKV-induced neurological disease. In contrast, while CD4+ and CD8+ T cells infiltrate into muscle tissues in RRV-infected mice, innate immune components, such as complement component 3 (C3), as opposed to the adaptive immune response, appear to be a major driver of RRV-induced disease [76,117].

## 4. Medical Countermeasures Against Alphavirus-Induced Disease

Table 2 summarizes the currently approved and potential countermeasures to combat Old World alphavirus-induced disease.

### 4.1. Preventative Strategies Against Old World Alphaviruses

As all alphaviruses are transmitted by mosquitoes, controlling vectors is one of the most important strategies for combatting alphavirus-induced disease. Different vector control approaches include insecticides, removing vector habitats near urban areas, surveillance, and education [132]. In 2017, the World Health Assembly approved the Global Vector Control Response developed by the World Health Organization (WHO). This strategic plan tackles vector-borne diseases, including CHIKV, through effective, locally adapted, sustainable vector control [132]. In 2022, the WHO launched the collaborative Global Arbovirus Initiative, which aims to improve the surveillance, prevention, and control of arboviruses and specifically focuses on countries with the heaviest burden and multiple circulating arboviruses [133].

Another strategy for preventing alphavirus-induced disease, and arguably the most crucial approach for preventing neurological disease cases, is vaccines. In late 2023, the live attenuated CHIKV vaccine VLA1553 was approved by the Food and Drug Administration for persons 18 years and older [134]; similar approval was granted in Canada and Europe in mid-2024 [135,136], and regulatory review is currently ongoing in the United Kingdom and Brazil. The CHIKV vaccine encodes the La Reunion strain of CHIKV lacking 61 amino acids in nsP3, which are essential for infection [137]. In the phase III clinical trial, neutralizing antibody titers were induced to protective concentrations and persisted through day 180 [118]. However, this vaccine is not approved for young children, who are one of the most vulnerable populations, especially to neurological disease manifestations [38]. Two clinical trials targeting children are currently in progress: one for adolescents aged 12 to 17 (NCT04650399) and one for young children aged 1 to 11 (NCT06106581). Furthermore, the CHIKV vaccine is not yet available in endemic countries, heavily underscoring the need for additional alphavirus treatment options for affected regions.

Due to their narrow geographic distribution, only limited data on vaccine development efforts for MAYV and ONNV are available. Several vaccines for MAYV are in preclinical development [9], including a live attenuated vaccine [138], adenoviral vector vaccines [57,139,140], and, more recently, particle-like vaccines [141]. At the publishing of this article, none are currently in the clinical trial phase. However, with the spread of vectors and detection of both viruses in *Ae. aegypti*, the close phylogenetic relationship of CHIKV and MAYV [142] may allow for cross-protection against MAYV infection with the CHIKV vaccine [57]. Cross-protection against MAYV has been reported in mouse models, although the mice used were deficient in type I IFN and thus immunocompromised [57]. No studies investigating cross-protection of VLA1553 with other alphaviruses have yet been reported. Unlike MAYV, as of 2024, no vaccines are in development for ONNV. Like MAYV, since ONNV and CHIKV are also closely related [3], the possibility of cross-reactivity with CHIKV vaccines has been raised [143]; however, the feasibility of the CHIKV vaccine cross-reacting with other Old World alphaviruses remains a hypothesis and requires formal evaluation.

RRV has no approved vaccine, but phase I/II and phase III vaccine studies have been completed. A phase III clinical trial investigating the safety and immunogenicity of a Vero-derived whole-cell RRV vaccine found that 91% of participants 16 to 59 years old and 76% of participants aged 60 years or older achieved strong RRV-specific neutralizing antibody titers [119]. However, the low incidence of RRV in Australia precluded the assessment of vaccine efficacy. As of December 2024, no other RRV vaccine approvals or trials are ongoing.

### 4.2. Treatments for Old World Alphavirus Infection

As of writing this review, while no post-exposure treatments have been approved for any Old World alphaviruses, several antiviral drugs are under investigation (Figure 1) [121,122,123]. For CHIKV, 3-methyltoxoflavin is a potent protein disulfide isomerase inhibitor that impedes E2-E1 folding and assembly in CHIKV but not in other alphaviruses [120]. Chloroquine has been shown to inhibit multiple viruses in vitro, including SINV, SFV, CHIKV, and MAYV by targeting viral uncoating [124,125]. However, a clinical trial studying the effect of chloroquine against acute CHIKV infection did not provide significant benefit [125].

Monoclonal antibodies (mAbs) for both prophylaxis and treatment against alphaviruses have been studied extensively and are especially promising due to their cross-neutralizing potential [3]. Multiple anti-CHIKV mAbs targeting the E2 glycoproteins have been evaluated, with some conferring protection in mouse models [20,126,127,128]. Interestingly, two anti-CHIKV mAbs have been shown to protect against not only CHIKV infections but also against MAYV, ONNV, and RRV [126,129]. A lipid nanoparticle-encapsulated messenger RNA (mRNA-1944) developed by Moderna showed promise in a phase I clinical trial [130]; however, current plans for phase II trials are unknown [35].

Because no approved treatments for alphavirus-induced disease are available, treatment is limited to symptomatic care [144]. For neurological complications following CHIKV infection, symptomatic care is based on the physicians’ assumption that the neuropathological process is virus or host-immune driven. When the assumed mechanism is virus driven, approaches that mitigate symptoms but do not directly combat the virus, such as non-steroidal anti-inflammatory drugs (NSAIDs) to relieve pain and reduce fever, are used [3,9,20,35]. However, immunosuppressives are used when the mechanism is assumed to be immune mediated, such as with processes like GBS [38].

### 4.3. Considerations for Treating Neurological Disease Caused by Old World Alphaviruses

The nature of neurological disease induced by Old World alphaviruses presents a significant challenge for developing potential treatments. Infection with Old World alphaviruses is usually not diagnosed until patients present with neurological complications; at this point, replicating virus, and often even viral RNA, are generally no longer detectable. Antivirals, mAbs, and drugs targeting virus entry and replication are, therefore, not likely to be effective solo treatment options once neurological symptoms are present. Antiviral treatments alone may be limited in alleviating neurological symptoms, as pathology is primarily immune driven [12], and a combination of antivirals and host-directed interventions may be a more effective treatment approach. Antivirals can target viral replication, which may reduce viral load and modulate the immune response to mitigate CNS damage and neurological symptom development. In theory, treatments that target the immune response represent a promising approach for treating neurological complications. For example, treatment for GBS, an immune-mediated neurological condition occasionally induced by CHIKV infection, involves eliminating harmful antibodies through plasmapheresis or administering intravenous immunoglobulin from healthy patients that suppresses the immune system [12]. However, developing effective treatments that can be targeted to alleviate and stop neurological damage requires an increased understanding of host-mediated mechanisms behind Old World alphavirus-induced neurological disease. Finally, once neurological symptoms are unmanageable enough for patients to seek medical help, the damage to the CNS is usually severe and difficult to reverse [38,48,53]. Therefore, while efforts to develop post-exposure treatments should continue to be pursued, preventives, such as vaccines, likely represent the most effective medical countermeasures to combat alphaviruses and should be made available to vulnerable populations in endemic countries.

## 5. Conclusions

As global temperatures rise and mosquito vectors spread to new geographical territories, alphaviruses are a progressive public health threat. Cases of neurological disease induced by CHIKV infection are increasingly being identified, and other Old World alphaviruses have the potential to cause similar atypical disease manifestations. Because neurological symptoms often do not appear until viremia is no longer detectable in the patient, supporting the development of effective preventative measures is especially critical. While the United States, Canada, and Europe have approved a live-attenuated CHIKV vaccine, it is not yet approved or available in endemic countries, highlighting the importance of making preventatives accessible to those most in need. Furthermore, as the immune response is strongly implicated as the primary driver of Old World alphavirus-induced neurological disease, therapies that target that aberrant immune response are likely to be more successful in mitigating damage to the CNS than virus-targeting approaches. However, while the immune response to alphaviruses can be pathogenic, it is also critical for protecting the patient by clearing the infection and preventing persistent viral RNA from reactivating. Therefore, it is of utmost importance to develop animal models of neurological disease induced by CHIKV, ONNV, MAYV, and RRV that better reflect the disease process seen in humans in conjunction with the already well-characterized mouse models of SINV and SFV to determine which pathogenic immune factors are best to target.

## Figures and Tables

**Table 1:** Summary of case reports of neurological disease for Old World alphaviruses.

Location, Year, Ref	Age(s), n Sex	Neurological Symptoms/Imaging	Laboratory Testing	Diagnosis	Treatment, Outcome
CHIKV					
Réunion Island, 2006 [36,37]	51, F	Bilateral facial palsy Quadriparesis Global areflexia	Serum positive for anti-CHIKV IgM	GBS	IV IgG, partial recovery
Réunion Island, 2007 [36]	60, M	Distal hypoesthesia Left facial palsy Quadriparesis	Serum positive for CHIKV IgM 7 dpa Serum positive for CHIKV by RT-PCR	GBS	
Switzerland, 2014 (traveler acquired from Tahiti) [38]	74, M	Hypesthesia Partial paralysis alpha coma	Serum positive for anti-CHIKV IgM/IgG	GBS	None
California (traveler acquired from Tonga), 2014 [39]	57, M	Reduced level of consciousness tonic-clonic seizure	Serum positive RT-PCR Serum positive for anti-CHIKV IgM/IgG	Encephalitis	Supportive care, Anti-epileptics, Partial Recovery
Honduras, 2015 [40]	0–1, 14 M and F	Bulging fontanelle Mild brain edema Seizures	CSF and serum RT-PCR positive for CHIKV	Meningo-encephalitis	Symptomatic care with NSAIDS, 2 died
Honduras, 2015 [41]	28–85, 5 M and 2 F	Stupor Status epilepticus periventricular lesions	Anti-CHIKV IgM negative	Encephalitis	IV acyclovir, 2 F died, others recovered
India, 2016 [42]	55, M	Altered sensorium Hyperintense lesion in the splenium	Serum and CSF RT-PCR positive for CHIKV	Mild encephalitis with splenial lesion	IV fluids, Recovered
Korea (traveler acquired from Cambodia), 2023 [43]	40, M	Vertigo Confusion Ataxia	Serum was positive for anti-CHIKV IgM 5 dpa	Encephalitis	IV methylprednisolone, recovered
India, 2024 [44]	12, 1 M	Decline in consciousness White matter brain lesions	CSF and serum RT-PCR positive for CHIKV	Encephalitis	Intravenous IgG
MAYV					
French Guiana, unknown date [45]	47, M	Confusion Phonophotophobia Kernig and Brudzinski positive	IgM MAYV seroconversion	Meningo-encephalitis	Unknown, Recovered
RRV					
Papua New Guinea, 1980 [46]	27, M	Polymorphonuclear leucocyte response in CSF	Serum RRV antibody titer positive 7 dpa	Acute encephalitis	Died
Australia, 1993 [33]	40, M	Head and neck stiffness	Serum positive for anti-RRV IgM	Possible meningitis	Unknown, Partial recovery
Central Australia, 1997 [47]	33, M	Confusion Personality changes Speech difficulties	Serum positive for anti-RRV IgM 8 dpa and IgG 90 dpa	Viral meningo-encephalitis	Acyclovir Ceftriaxone phenytoin

Abbreviations: chikungunya virus, CHIKV; immunoglobulin M, IgM; immunoglobulin G, IgG; Guillain–Barré syndrome, GBS; cerebrospinal fluid, CSF; real-time polymerase chain reaction, RT-PCR; non-steroidal anti-inflammatory drugs, NAIDS; Mayaro virus, MAYV; Ross River virus, RRV; days post-admission, dpa.

**Table 2:** Countermeasures for neurological disease caused by Old World alphaviruses.

Countermeasure	Alphavirus	Mechanism/Target	Status	Refs.
Prevention				
Attenuated vaccine	CHIKV, evidence of cross reactivity	Attenuated nsP3 CHIKV	Approved in US, CA, and EU	[118]
Inactivated whole-virus vaccine	RRV	Formalin- and UV inactivated	Phase III trial completed	[119]
Virus-Directed Treatments				
3-methyltoxoflavin	CHIKV	Inhibits E2-E1 folding	In vitro studies completed	[120]
Chloroquine	SINV, SFV, CHIKV, MAYV	Inhibits viral uncoating and the type I interferon response	No benefit in Phase II trial	[121,122,123,124,125]
CHIKV-265	CHIKV, MAYV, ONNV, RRV	Neutralizing antibody targeting E2	Pre-clinical studies	[126,127,128,129]
mRNA-1944	CHIKV	Encodes human anti-CHIKV to elicit neutralizing antibodies	Phase I trial completed	[130]
Host-Directed Treatments				
Supportive care	Pan-alphavirus	Hydration, rest	In Use	[38]
NSAIDs	Pan-alphavirus	Pain reliever and fever reducer	In Use	[38]
Immunosuppressives	Pan-alphavirus	Suppress immune response to limit neuropathology	In Use	[38]
Intravenous IgG	Pan-alphavirus	Suppresses immune system to combat GBS	In Use	[131]
Plasmapheresis	Pan-alphavirus	Remove harmful antibodies involved in GBS	In Use	[131]

Abbreviations: chikungunya virus, CHIKV; non-structural protein 3, nsP3; Ross River virus, RRV; ultraviolet, UV; non-steroidal anti-inflammatory drugs, NAIDS; immunoglobulin G, IgG; Guillain–Barré syndrome, GBS; Sindbis virus, SINV; Semliki Forest virus, SFV; Mayaro virus, MAYV; O’nyoug-nyoug virus, ONNV; messenger ribonucleic acid, mRNA.
